# Longevity and relationships with children: the importance of the parental role

**DOI:** 10.1186/1471-2458-9-351

**Published:** 2009-09-18

**Authors:** Maria-Victoria Zunzunegui, François Béland, Maria-Teresa Sanchez, Angel Otero

**Affiliations:** 1 Département de Médecine Sociale et Préventive, Université de Montréal, Montréal, Canada; 2 Département de l'Administration de la Santé, Université de Montréal; Codirector - SOLIDAGE, Lady Davis Institute, Jewish General Hospital of Montreal, Montréal, Canada; 3 Departamento de Medicina Preventiva, Universidad Autónoma de Madrid, Madrid, Spain. Hospital Universitario La Paz, Madrid, Spain

## Abstract

**Background:**

Social networks predict longevity across societies but specific mechanisms are largely unknown. The aim of this work was to examine the role of children in the longevity of elderly men and women in a cohort of community dwelling elderly people in Spain.

**Methods:**

The data were taken from the "Aging in Leganes" cohort study with 15 years of follow-up. The baseline population was an age- and sex-stratified random sample of community dwelling people over 65 living in Leganés (Madrid) in 1993. Poor relationship with at least one child, emotional support and the perceived roles elders play in the lives of their children, extended family, spouse and friends were assessed at baseline. Cox proportional hazards models were fit to investigate the effects of social roles variables on longevity, adjusting for a wide range of socioeconomic, behavioural and health covariates.

**Results:**

In the fully adjusted model, having a poor relationship with at least one child increased mortality by 30%. Elderly persons who felt their role in their children's lives was important (HR = 0.70; 95% CI 0.54; 0.91) had a lower mortality risk than those who felt they played a small role. Feeling loved and listened to by one's children did not have an effect on survival. Maintaining an important role in the extended family was also significantly associated with survival.

**Conclusion:**

In this Mediterranean population, maintaining an important role in the lives of one's children is associated with survival. Functions of social networks related to meaning of life and different forms of social support may have important effects on mortality, and these functions may vary across cultures according to family norms and values.

## Background

Social networks predict longevity across societies [1-7]. Pioneer studies have examined the effects of social ties on survival using overall measures of social connectedness [2,3,7,8]. More recent studies from North America, Europe, Japan and Australia have found positive associations between longevity and social integration in the community [9-13], being married [12,14] and having friends [6,12,15]. The impact of relationships with one's adult children on mortality has rarely been examined. Silverstein & Bengston [16] in California reported that children's support had no general effect on survival, but that affection from children could attenuate the impact of recent widowhood on mortality. A comparative study of the role of social networks in the longevity of Swedish and American elders found that children were important for the longevity of Swedish elderly people, whereas friends and spouse were more salient for the elderly in the U.S., despite the fact that the frequency of contacts with children was similar in the two countries [5]. An Australian study reported negligible effects of social networks with children on their parents' longevity [1]. Thus, research on aspects of the relationships of elderly people with their children and survival has produced conflicting results across the few regions of the world where this research has been undertaken.

These conflicting results are also found when research is extended to include health outcomes. Thus, European studies have shown positive effects of relationships with children on elderly parent's health [17-19] but results were not replicated in two Canadian samples using similar measurement instruments [20]. A study in the United States examining the role of social support in disablement found significant associations for support from spouse and friends, but no association between support from children and risk of disability [21].

Berkman & Glass have proposed a theoretical model according to which culture, political context and social change determine social networks which, in turn, influence health via psychological and physiological pathways [22]. We propose an additional mechanism by which social relations may influence survival status: reinforcement of social roles through recognition of the marital, parental, familial or friendship role by spouse, children, relatives and friends. We argue that in cultures where filial obligation prevails in a context of intergenerational interdependence, reinforcement of the parental role will have beneficial effects for elderly parents' survival, independently of other components of social networks (such as spouse, friends and community integration). In addition, in familial societies such as those prevailing in the Mediterranean region, the recognition of the role of the elderly by the younger members of the family may also contribute to their survival.

Many studies on social relationships and health have used the social exchange theory to explain the dynamics of these relationships [23-27]. According to this theory, social relationships are governed by a norm of reciprocity [28]. Studies on social relationships and longevity based on social exchange theories have attempted to explain how the elderly person receives social support and how this type of support affects longevity. However, few studies have examined the importance of mutual dependence for the elderly themselves. Mutual dependence builds cohesion in primary relationships such as those that tend to be found in families [28]. For elderly people, approval, affirmation and respect may be highly valued; recognition of experience and respect from their family will give meaning to life. Krause proposes that meaning to life is linked to receiving emotional support, anticipated support and negative interactions[29]. He also proposes that playing a role in a valued social institution could foster the sense of meaning to life[29]. We add that for many countries and cultures the family is a valuable social institution and that playing a role in the family and, particularly, a parental role will increase meaning to life and indirectly will increase longevity. In this paper, we present the results of a study with 15 years of follow-up data, focusing on the role of social networks with children on longevity. The aim of this work was to examine the extent to which reinforcement of the parental social roles through recognition by children is associated with longevity of elderly men and women in a cohort of community dwelling elderly population in Spain.

## Methods

The data were taken from the "Aging in Leganes" cohort study (1993-2008) with 15 years of follow-up. Its main objective was to assess the impact of social relationships on the physical and mental health of a community dwelling elderly population. The study population was an age- and sex-stratified random sample (n = 1560) of community dwelling people over age 65 (range 65-101) living in Leganés, a suburban municipality located 8 kilometres outside Madrid. The response rate was 82% (n = 1283). The sample was representative of the elderly people in Leganés, whose age, sex, marital status and education resemble those of the Spanish elderly population as a whole. In 1993, only 2% of the elderly in Spain were institutionalized. Close to 93% of the participants had children and 45% of them co-resided with them. Baseline data were collected in 1993, through home interviews and a medical exam. For this paper, the sample consisted of participants who had children (n = 1186), were able to answer the questionnaire on social network characteristics entirely by themselves, and for whom complete data were available on all covariates (n = 903). Those with children and incomplete data (n = 283) had significantly higher mortality, were older, had less education and more cognitive impairment than the complete data sample (n = 903).

### Mortality

Deaths were determined by computer linkage to the National Death Registry, upon authorization of the Ministry of Health, and using the National Identity Number, the first name and two last names, as is customary in Spain. All deaths occurring between study entry (April 1993-November 1993) and 31 August 2008 were ascertained. There were 863 (67.3%) deaths among the 1283 participants older than 65 years of age in the 1993 baseline survey and 579 deaths (64.1%) in this study sub-sample.

### Social networks

Based on our literature review of the effects of social networks on health status, two dimensions of networks were measured in 1993: social support and social roles. Our main independent variable in this paper is the parental role but social support and social roles measurements were made for each of four types of networks: children, extended family (siblings, nephews and nieces, grandchildren), spouse and friends. Seven questions were asked in relation to each type of tie. The elderly participants were asked how frequently they a) felt loved and cared for; b) felt listened to; c) felt criticised; d) would like to confide more in their significant others; e) helped them; f) felt they played an important role in their lives; g) felt useful to them. Possible answers were 'never' (1 point), 'sometimes' (2 points), 'frequently' (3 points), and 'always' (4 points). The first two questions were taken from the MacArthur Community Study (USA) [30]. We added the last five questions to measure aspects of conflict and social roles. A factor analysis (principal axis factoring, varimax rotation) was performed. Two factors were found: Questions a) and b) scored in factor one, which was subsequently named 'received emotional support'; questions e), f) and g) scored on a second factor, which was named 'social role" To build scales, the raw item scores of the questions in each factor were added and averaged. Questions c) and d) had low loadings (< 0.40) on both factors and therefore, were excluded. Cronbach's alpha varied between 0.74 and 0.83 for received emotional support and between 0.70 and 0.74 for social role. These two scales have been used in our previous work [31,32].

The question on criticism (negative support) was not well accepted since elderly people said that children are not supposed to criticize their parents or relatives. Thus, to measure conflict in the relationship with children, we used participants' reports of the number of children with whom they had a good relationship. This number was subtracted from the total number of children to estimate the number of those with whom they did not have a good relationship. An indicator variable was created to distinguish those who did not have a good relationship with at least one child.

### Potential confounders

Sociodemographic variables included age, sex and education. Education was categorized as: unable to read and write, no schooling but can read and write, incomplete primary, and complete primary education or more. Health status was measured by the number of chronic conditions, cognitive function and activities of daily life (ADL) disability. Chronic conditions were ascertained by asking the elderly person if he/she had been diagnosed by a doctor with any of a list of 11 chronic problems (hypertension, heart disease, circulation problems, stroke, diabetes, chronic respiratory problems, arthritis, cancer, Parkinson's disease, genitourinary and gastrointestinal problems). Cognitive function was ascertained using the *Prueba Cognitiva de Leganes *(PCL) [33,34], a screening test for dementia in populations with low levels of education. For descriptive purposes, the cognitive score was dichotomized at the cut-off for dementia, but used as a continuous variable in multivariate modelling. ADL disability was ascertained by asking individuals if they were able to carry out 8 activities of daily living alone, with help or not at all. Based on this information, a dichotomous variable was created with the value of 1 if the person was unable to carry out one or more activities without help and zero otherwise. Smoking was categorized as never smoked, ex-smoker or currently smoking. Physical activity was coded as a dichotomous variable to distinguish those reporting light or no exercise from those reporting moderate or vigorous exercise.

Characteristics of social relationships with extended family, spouse and friends and community activities were included as potential confounders of the association between children networks and mortality, since our aim was to test if an important parental role is associated with better survival, independently of family relationships and community activities. Family roles and support were coded as explained above. An additional category was added for those without spouse or without friends. Social activities in the community were ascertained by adding the number of monthly visits to a church, a senior citizens center or the local square (equivalent to food market or neighbourhood shopping center). The cumulative number of social activities was categorized in quartiles.

### Statistical analysis

Univariate Cox proportional regression models were used for each covariate to estimate hazard ratios and their 95% confidence intervals. The assumption of proportional hazards was assessed by regressing the scaled Shoenfeld residuals against the log of time and testing for zero slope [35]

Hazard ratios (HR) for characteristics of children networks were estimated using multivariable Cox regression. We controlled for socio-demographic variables (age, sex and education), health status (number of chronic conditions, cognitive function score and ADL disability), social activities in the community (monthly frequency of attending religious services, social community centers for the elderly, neighbourhood square), and spouse, family and friends support and roles. The functional form of the mortality hazards of continuous variables was explored (age, number of chronic conditions, cognitive score) and linearity was accepted for all of them. For social network variables we used categories (tertiles) in order to illustrate the presence or absence of possible thresholds. Variables were included sequentially in the model: first socio-demographics; second, health and disability status indicators and third, social network variables. All interactions of social network variables with sex were tested given the conflicting results of research on gender differences in the relationships between social networks and mortality [36]. SPSS version 16 was used for factor and survival analysis.

### Ethical aspects

This study has been approved by the Ethics Committee for Clinical Research of the IX Health Region of Madrid (Hospital Severo Ochoa de Leganés) and by the Ethics Committee for Research at the Universidad Autónoma de Madrid.

## Results

The mean age of the analytical sample was 75.5 ± 7.3 years, 64% were married and level of education was low: 15% were illiterate and only 20% had finished primary education. The prevalence of ADL disability reached 24% and 78% had two or more chronic conditions.

### Univariate survival analysis of characteristics of children networks

Table 1 shows the distribution of the characteristics of children networks and their univariate hazard ratios. Conflict in the relationship with children and the perceived importance of the role these elders played in their children's lives ("parental role") were associated with survival. Those who had a poor relationship with at least one child had a higher mortality risk than those who reported good relationships with each child. The association between parental role and mortality showed a dose response trend. Those who reported that they played an important parental role for their children had the lowest mortality risk, and those who considered their role somewhat important had a lower risk than those who perceived to play a small role. Children's emotional support at baseline was not associated with mortality.

**Table 1:** Children's networks characteristics: Univariate Hazard Ratios and 95% CIs for mortality at 15 years

	**N**	**% deaths**	**HR**	**95% CI**	**p-value**
Poor relationship with at least one child					< 0.001
*No*	793	62.3	1.0		
*Yes*	110	77.3	1.58	(1.26;1.99)	
Parental role					< 0.001
*Small*	259	78.4	1.0		
*Some*	335	64.2	0.65	(0.59;0.79)	
*Important*	309	52.1	0.47	(0.38;0.58)	
Emotional support from children					0.395
*Low*	216	66.2	1.0		
*Medium*	285	66.0	0.98	(0.79;1.22)	
*High*	402	61.8	0.86	(0.70;1.06)	

### Univariate survival analysis of sociodemographic, health and social networks potential confounders

Table 2 shows the distribution of the potential sociodemographic and health confounders with their univariate hazard ratios and 95% confidence intervals. As expected, mortality risk increased with advancing age and was higher in men, widows, and those with little education. Cognitive function, ADL disability, and chronic conditions were all significantly associated with survival in this univariate analysis. Physical activity and never having smoked were also associated with survival.

**Table 2:** Univariate Hazard Ratios and 95% CIs for mortality by sociodemographic and health characteristics

**Characteristic**	**N**	**% deaths**	**Crude HR**	**95% CI**	**p-value**
Age					< 0.001
*65-69*	229	35.8	1.0		
*70-74*	215	54.0	1.74	(1.31;2.30)	
*75-79*	191	71.7	2.83	(2.15;3.73)	
*80-84*	150	87.3	4.55	(3.44;6.01)	
*85+*	118	95.8	7.91	(5.91;10.58)	
Sex					0.001
*Men*	476	71.2	1.53	(1.30;1.81)	
*Women*	427	56.2	1.0		
Marital status					< 0.001
*Widow*	316	71.5	1.46	(1.23;1.72)	
*Married*	587	60.1	1.0		
Education					0.035
*Illiterate*	137	72.3	1.46	(1.11;1.91)	
*At least read and write*	270	63.7	1.16	(0.91;1.48)	
*Incomplete primary*	317	63.7	1.07	(0.85;1.36)	
*Primary and more*	179	59.2	1.0		
Number of chronic diseases					0.022
*Zero or one*	153	56.2	1.0		
*Two or three*	365	64.4	1.25	(0.98;1.60)	
*Four to five*	277	64.6	1.25	(0.97;1.62)	
*Six and more*	108	73.1	1.62	(1.19;2.20)	
Cognitive function score					< 0.001
*<23*	211	78.7	1.78	(1.49;2.14)	
*≥24*	692	59.7	1.0		
ADL disability					< 0.001
*Yes*	220	79.1	1.85	(1.55;2.21)	
*No*	683	59.3	1.0		
Smoking					0.001
*Never*	520	59.2	1.0		
*Ex-smoker*	287	71.4	1.38	(1.15;1.64)	
*Current Smoker*	95	68.4	1.30	(0.99;1.70)	
Physical exercise					< 0.001
*Vigorous*	23	21.7	0.15	(0.06;0.36)	
*Moderate*	632	60.3	0.57	(0.48;0.68)	
*Light*	245	77.6	1.0		

The results of the univariate analysis for social network characteristics other than children networks are shown in table 3. Similar results were obtained for the role of elders in the lives of their extended family ("family role") and in their spouse's life ("marital role"), and for emotional support received from the spouse. Those without a spouse had a mortality risk similar to those who did not feel important for their spouse. Playing an important role in the lives of friends ("friendship role") was associated with lower mortality, but mortality in those who did not consider they played an important role in this respect was similar to mortality in those who had no friends. Emotional support from friends was significantly associated with survival. Also the frequency of activities in the community was associated with survival, but in a non linear fashion.

**Table 3:** Univariate Hazard Ratios and 95% CIs for mortality by social network characteristics (other than children networks)

**Characteristic**	**N**	**% deaths**	**Crude HR**	**95% CI**	**p-value**
Family role					< 0.001
*Small*	312	74.0	1.0		
*Some*	353	58.4	0.62	(0.51;0.75)	
*Important*	238	59.7	0.65	(0.53;0.80)	
Emotional support received from family					0.128
*Low*	301	67.8	1.0		
*Medium*	401	60.6	0.83	(0.69;1.00)	
*High*	201	65.7	0.96	(0.77;1.20)	
Marital role					< 0.001
*Small*	124	73.4	1.0		
*Some*	121	65.3	0.78	(0.57;1.05)	
*Important*	328	53.0	0.57	(0.45;0.74)	
*No spouse*	325	71.4	1.02	(0.80;1.30)	
Emotional support received from spouse					0.003
*Low*	102	57.8	1.0		
*Medium*	239	57.3	0.94	(0.69;1.28)	
*High*	234	63.7	1.13	(0.83;1.52)	
*No spouse*	325	71.4	1.50	(1.13;2.00)	
Friendship role					0.003
*Small*	166	67.5	1.0		
*Some*	129	57.4	0.73	(0.55;0.98)	
*Important*	149	54.4	0.69	(0.52;0.92)	
*No friends*	459	68.0	1.01	(0.82;1.26)	
Emotional support received from friends					0.026
*Low*	120	56.7	1.0		
*Medium*	81	60.5	1.07	(0.74;1.55)	
*High*	245	61.2	1.17	(0.88;1.56)	
*No friends*	457	68.3	1.40	(1.08;1.82)	
Activities in the community					< 0.001
*First quartile*	261	74.3	1.0		
*Second quartile*	236	60.6	0.67	(0.54;0.83)	
*Third quartile*	200	53.0	0.54	(0.43;0.69)	
*Fourth quartile*	206	66.0	0.74	(0.59;0.92)	

### Multivariate survival analysis

The associations between children networks and mortality are presented in Table 4. Three models were fitted, and the results from the univariate analyses were confirmed after controlling for all potential confounders. Estimates of hazard ratios were stable across models. In the fully adjusted model, having a poor relationship with at least one child was associated with a 30% increase in the hazard ratio for mortality (HR = 1.30; 95% CI 1.02; 1.67). Those elderly persons who felt they played an important role (HR = 0.71; 95% CI 0.55; 0.91) or some role (HR = 0.91; 95% CI 0.73; 1.12) for their children had a lower risk of mortality than those who felt they had a small role in their children's lives. Emotional support and feeling loved and listened to by one's children did not have a significant effect on survival. None of the interactions of social networks by sex attained statistical significance, indicating that the impact of children networks on longevity is similar in elderly men and women.

**Table 4:** Adjusted Hazard Ratios and 95% CIs for mortality at 15 years by characteristics of children networks

	**Poor relationship with at least one child**	**Parental role**		**Emotional support received from children**	
		
		**Some vs Small**	**Important vs Small**	**Medium vs low**	**High vs low**
	**HR** **(95% CI)**	**HR** **(95% CI)**	**HR** **(95% CI)**	**HR** **(95% CI)**	**HR** **(95% CI)**

**Model 1**					
adjusted for age, sex, education	1.43 (1.12;1.82)	0.73 (0.60;0.89)	0.60 (0.48;0.75)	1.13 (0.90;1.43)	1.05 (0.84;1.30)

**Model 2**					
Model 1+ number of chronic conditions, cognition and ADL disability, physical activity and smoking	1.33 (1.04;1.69)	0.82 (0.67;1.00)	0.67 (0.52;0.83)	1,09 (0.86;1.38)	1.01 (0.81;1.26)

**Model 3**					
Model 2 + marital support and usefulness, family support and usefulness, Friends' support and usefulness, frequency of community activities	1.30 (1.02;1.67)	0.91 (0.73;1.12)	0.71 (0.55;0.91)	1.13 (0.90;1.43)	1.07 (0.86;1.33)

### Complete final multivariate model

**Figure 1:**
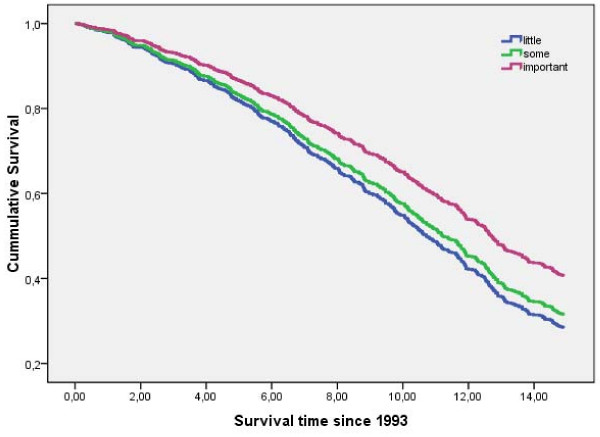
**Fifteen year survival by importance of parental role**.

Table 5 shows the results for the final model, including all significant variables. An important parental role (Figure 1) and having good relationships with all children were associated with survival. Of the remaining social network characteristics, only role in the extended family was significant once the children network variables were included in the model. The support of spouse and friends and marital role were not significantly associated with survival. As expected, smoking and lack of physical activity were significant mortality risk factors, and the magnitude of their association with mortality was similar to the hazard ratios for having an important parental role and having good relationships with all children.

**Table 5:** Multivariate hazards ratios: Children networks and all significant social network, health, health habits and sociodemographic variables

	**HR**	**95% CI**	**p-value**
Poor relationship with at least one child			0.034
*No*	1.0		
*Yes*	1.30	(1.02;1.66)	
Parental role			0.021
*Small*	1.0		
*Some*	0.91	(0.73;1.13)	
*Important*	0.70	(0.54;0.91)	
Emotional support from children			0.662
*Low*	1.0		
*Medium*	1.12	(0.89;1.43)	
*High*	1.05	(0.84;1.32)	
Family role			0.007
*Small*	1.0		
*Some*	0.72	(0.58;0.89)	
*Important*	0.89	(0.69;1.14)	
Sex			0.010
*Women*	1.0		
*Men*	1.40	(1.08;1.82)	
Education			0.080
*Literacy*	1.0		
*Illiteracy*	1.23	(0.98;1.55)	
Age (one additional year)	1.09	(1.07;1.10)	< 0.001
Cognitive function score (one additional unit)	0.97	(0.95;0.99)	0.005
Comorbidity (one additional chronic disease)	1.08	(1.03;1.13)	0.002
Smoking			0.027
*No smoker*	1.0		
*Ex-smoker*	1.36	(1.05;1.77)	
*Smoker*	1.50	(1.08;2.08)	
Physical activity			0.002
*Light*	1.0		
*Vigorous*	0.23	(0.09;0.56)	
*Moderate*	0.83	(0.68;1.00)	

## Discussion

Our results indicate that relationships with children have strong and important effects on elderly parents' survival and that these effects are related with the parental role and absence of conflict with children. Emotional support from children does not seem to have an independent effect on mortality. Playing an important role in the extended family is also associated with survival. However, neither emotional support from family, spouse or friends nor marital or friendship roles are associated with survival once the effect of parental and family roles are taken into account. Social activities in the community are not independently associated with survival once family relationships are considered.

We have previously shown that 6-year survival in this Spanish population was higher among elders with community involvement through social participation, those engaged in multiple roles with significant others, and those with a confidant [10]. Here, we extend the previous analyses to 15 years of follow up. The large number of deaths makes it possible to examine the functions of each type of network - children, relatives, spouse, and friends - as well as social activities in the community, and particularly to make a quantitative assessment of the important role of children in the survival of elderly people.

As far as we know, this is the first study that has examined aspects of the parent-child relationship other than frequency of contact and social support on elderly people's survival. Our results illustrate two points: research on social networks and mortality needs to 1) examine the associations between mortality and specific social network components instead of relying on a summative index of total social networks and 2) consider functions of social networks other than social support. Our results support Krause's hypothesis on the beneficial effects of social relationships by fostering a sense of meaning to life [29] by testing it in a country where family and particularly children are central to elderly people's life.

Studies examining the role of children in their parents' survival have produced conflicting results, which may be due to cultural differences in intergenerational relationships and in the role of elders in family life. The effects of social networks of children and relatives were not significantly related to 10-year survival in a recent Australian study that used a measurement instrument similar to ours, which was also derived from Glass et al [37]. However, in the Australian study the protective effects of friends and confidants were large. In Denmark, a study carried out on a cohort of twins reported significant effects for relationships with co-twin, spouse and friends, but no effects of children networks on parental mortality risk were observed [6]. Children's impact on parents' longevity is likely to be more salient in societies where contacts tend to occur on a daily basis and where co-residence is frequent. As many as 40% of the elderly over 65 live with their children in the South of Europe, whereas this percentage is less than 15% in Northern Europe and even lower in North America [38]. Institutionalization is still rare in Southern Europe; in Spain around 4% of all elderly people over 65 were institutionalized in 2007. Studies from Asia and Latin America have shown that co-residence remains the norm.

Our study has similarities with a 6-year follow-up study that examined the impact on mortality of subjective usefulness, assessed by a single question - "How do you evaluate your present usefulness to others and society?" - among the elderly population in a rural area of Japan [39]. The mortality hazard in those who rated their usefulness as low was 2.24 (95% CI 1.2; 4.3) times higher than in those who considered themselves quite useful. A study from California reported that feeling useful to friends and family was associated with lower mortality in a 7-year follow-up period [40].

Absence of conflict seems to increase survival[41]. To our knowledge, this is the first study that has attempted to measure the association between poor relationships with children and survival.

Some limitations need to be mentioned. Selection bias may be an issue since those who could not respond to the questionnaire on social networks had more cognitive impairment (mean cognitive score of 15 for excluded subjects compared with 25 for those included, the maximum score being 32). This limitation is common to studies which require self reported information on characteristics of social networks. Second, we had no information on diet or eating habits. Eating in the company of the family may increase the probability of achieving a balanced diet as opposed to the diet of elderly people living alone or living only with their spouse. Resource sharing and mutual help may increase well-being and longevity through adoption of healthy behaviours [27]. Lastly, the interactions between changes in social or health status and children networks were not tested in our relatively small sample.

The distribution of this elderly population in Leganes was similar in terms of age, sex, education, marital status and self-perceived health to the elderly Spanish population that participated in the first National Health Survey of Spain in 1993. Nevertheless, socioeconomic and cultural differences across the regions of Spain are large, although social exchanges between parents and children are extensive and similar to the situation in other Mediterranean countries [38]. Further research is needed to assess the applicability of these results to different cultures since the value of mutual and intergenerational dependence varies across cultures.

## Conclusion

The results of this study contribute to the evidence of the contribution of social engagement on valuable social institutions such as the family to a sense of meaning of life that leads to increase longevity. In this Mediterranean population, family and parental roles are important predictors of survival for elderly parents. Recognition by children and the extended family of the important role of elderly family members can reinforce their feelings of usefulness, mutual dependence, and belonging, acting on survival by physiological mechanisms which are not yet understood.

## Competing interests

The authors declare that they have no competing interests.

## Authors' contributions

MVZ conceived of the study, conducted the analysis of the data and wrote the first draft, FB contributed to the conception of the study and has given comments to all versions of the manuscript, MT Sanchez helped in the reference review and contributed to the data analysis and A Otero has contributed at all stages of the manuscript. All authors read and approved the final manuscript.

## Pre-publication history

The pre-publication history for this paper can be accessed here:


